# Effects of Glucose Availability on Expression of the Key Genes Involved in Synthesis of Milk Fat, Lactose and Glucose Metabolism in Bovine Mammary Epithelial Cells

**DOI:** 10.1371/journal.pone.0066092

**Published:** 2013-06-14

**Authors:** Hongyun Liu, Ke Zhao, Jianxin Liu

**Affiliations:** Institute of Dairy Science, Key Laboratory of Molecular Animal Nutrition, Ministry of Education, Zhejiang University, Hangzhou, China; Huazhong Agricultural University, China

## Abstract

As the main precursor for lactose synthesis, large amounts of glucose are required by lactating dairy cows. Milk yield greatly depends on mammary lactose synthesis due to its osmoregulatory property for mammary uptake of water. Thus, glucose availability to the mammary gland could be a potential regulator of milk production. In the present study, the effect of glucose availability on expression of the key genes involved in synthesis of milk fat, lactose and glucose metabolism *in vitro* was investigated. Bovine mammary epithelial cells (BMEC) were treated for 12 h with various concentrations of glucose (2.5, 5, 10 or 20 mmol/L). The higher concentrations of glucose (10–20 mmol/L) did not affect the mRNA expression of acetyl-CoA carboxylase, diacyl glycerol acyl transferase, glycerol-3 phosphate acyl transferase and α-lactalbumin, whereas fatty acid synthase, sterol regulatory element binding protein-1 and beta-1, 4-galactosyl transferase mRNA expression increased at 10 mmol/L and then decreased at 20 mmol/L. The content of lactose synthase increased with increasing concentration of glucose, with addition of highest value at 20 mmol/L of glucose. Moreover, the increased glucose concentration stimulated the activities of pyruvate kinase and glucose-6-phosphate dehydrogenase, and elevated the energy status of the BMEC. Therefore, it was deduced that after increasing glucose availability, the extra absorbed glucose was partitioned to entering the synthesis of milk fat and lactose by the regulation of the mRNA expression of key genes, promoting glucose metabolism by glycolysis and pentose phosphate pathway as well as energy status. These results indicated that the sufficient availability of glucose in BMEC may promote glucose metabolism, and affect the synthesis of milk composition.

## Introduction

As the main precursor for lactose synthesis, large amounts of glucose are required by lactating dairy cows. Compared with non-lactating dairy cows, the requirements of glucose are approximately fourfold greater in lactating cows [1]. Milk yield greatly depends on mammary lactose synthesis due to its osmoregulatory property for mammary uptake of water. Lactating mammary gland can consume up to 85% of the circulating glucose [2]. Thus, the availability of glucose to the mammary gland could be a potential regulator of milk yield. The metabolic products of glucose, such as ATP and NAPDH, are important factors in milk synthesis of dairy cows. The effects of infusions of different glucose levels on lactation performance and metabolic profiles have been extensively studied [3], [4]. Some studies have confirmed that milk yield curvilinear increased and milk protein concentration linear increased with increasing glucose levels [4], [5], whereas other trials have shown inconsistent results. With glucose infusion, Lemosquet et al. [6] and Al-Trad et al. [7] demonstrated no effect on milk yield, while Oldick et al. [8] observed a decrease in milk yield. These conflicting results may be ascribed to the inhibition or reduction of gluconeogenesis during glucose infusion [9], or the difference of diets providing post-ruminal supply of starch [3], [5].

However, little information is available on the effects and not clear what mechanisms would be for glucose availability in bovine mammary epithelial cells (BMEC) *in vitro.* Xiao and Cant found that at physiological glucose concentrations, phosphorylation by hexokinase (HK) exerts 80% of the control of glucose metabolism to lactose and CO_2_, and transport exerts the remaining 20% [10]. Our previous study further revealed that glucose concentration affects glucose uptake partly by altering the activity of HKs, and HK2 may play an important role in the regulation of glucose uptake in BMEC [11]. In the present study, BMEC were used to investigate the effect of glucose availability on expression of the key genes involved in synthesis of milk fat, lactose and glucose metabolism *in vitro*. These results would help supplement the nutritional manipulation theories of glucose in the mammary gland of lactating dairy cows.

## Materials and Methods

### Ethics Statement

In the study, animal experiment was approved by the Institutional Animal Care and Use Committee and conducted in accordance with the guidelines for the care and use of experimental animals at Zhejiang University.

### Culture and Treatment of Bovine Mammary Epithelial Cells

Mammary tissues were obtained from two healthy Holstein dairy cows at the middle stage of lactation. Tissues were minced into 1 mm^3^ pieces and incubated at 37°C in a water-saturated atmosphere of 95% air and 5% CO_2_. The procedures of purification and culture of BMEC were described previously [12]. Briefly, BMEC and fibroblast cells were separated with 0.25% trypsin and 0.15% trypsin plus 0.02% EDTA. The dispersed cells were seeded at the density of 5×10^4^ cells/ml in six-well culture plates in DMEM/F12 medium (Gibco, Grand Island, NY, USA). The basal medium was supplemented with 1 µg/ml hydrocortisone, 5 µg/ml prolactin, 5 µg/ml insulin, 5 µg/ml transferrin, 10 ng/ml epidermal growth factor (Sigma-Aldrich, St. Louis, MO), 1% streptomycin,1% penicillin, 1% glutamine, and 10% fetal calf serum (Sangon, Shanghai). Cells were incubated at 37°C in a water-saturated atmosphere containing 5% CO_2_. After BMEC covered 80% of the surface, the basal culture medium was changed to DMEM without glucose (Gibco, Grand Island, NY, USA) for 1 h, and then the medium was supplemented with various levels of D-glucose (2.5, 5, 10 or 20 mmol/L, Sigma, St Louis, MO, USA) for another 12 h to evaluate its effects on expression of key genes involved in synthesis of milk fat, lactose and glucose metabolism *in vitro*.

### The mRNA Abundance

Total RNA was extracted by Trizol Reagent (Invitrogen, Carlsbad, USA), and the first strand of cDNA was transcribed using a reverse transcription kit (Takara, Tokyo, Japan). The abundance of mRNA was detected by real-time quantitative PCR (ABI 7500, Applied Biosystems, Singapore) using SYBR PrimeScript™ reagent kit (Takara Biotechnology, China) in a reaction volume of 20 µL. For PCR amplifications, the primer pairs were designed and synthesized (Takara Biotechnology, China) as in Table 1. The amplification programs were as follows: 10 s of pre-denaturalization at 94°C, followed by 40 cycles of 5 s denaturation at 95°C, and 34 s annealing and extension at 60°C. The 2^−ΔΔCT^ (cycle threshold, CT) method was used to calculate the relative changes [13].

**Table 1:** Primers of the key genes involved in synthesis of milk fat and lactose for real-time polymerase chain reaction.

Gene	Gene No.	The primers	The length
ACC	NM-174224	5′-CATCTTGTCCGAAACGTCGAT-3′	101 bp
		5′-CCCTTCGAACATACACCTCCA-3′	
FAS	NM-001012669	5′-ACCTCGTGAAGGCTGTGACTCA-3′	92 bp
		5′-TGAGTCGAGGCCAAGGTCTGAA-3′	
DGAT	NM-174693	5′-CCACTGGGACCTGAGGTGTC-3′	101 bp
		5′-GCATCACCACACACCAATTCA-3′	
GPAT	NM-001012282	5′-GCAGGTTTATCCAGTATGGCATT-3′	63 bp
		5′-GGACTGATATCTTCCTGATCATCTTG-3′	
SREBP1	NM-001113302.1	5′-CCAGCTGACAGCTCCATTGA-3′	67 bp
		5′-TGCGCGCCACAAGGA-3′	
B4GALT	NM-177512	5′- GTTTGGATTTAGCCTACCTTA-3′	186 bp
		5′- TTCCCGATCACAGCATTT-3′	
LA	NM-174378	5′-GACTTGAAGGGCTACGG-3′	175 bp
		5′-TGTTGCTTGAGTGAGGG-3′	
β-actin	NM-173979	5′-GCCATGAAGCTGAAGATGAC-3′	244 bp
		5′-CCTTCTGCAGCTCAGATATG-3′	

### Lactose Synthase Content and Activities of Pyruvate Kinase and Glucose-6-phosphate Dehydrogenase

The content of lactose synthase (LS) was estimated using an enzyme linked immunosorbent assay kit (Huijia Biotechnology, Shanghai, China). The intensity of the product at 450 nm was directly proportional to the concentration of LS present in the samples. Activities of total pyruvate kinase (PK) and glucose-6-phosphate dehydrogenase (G6PD) were assayed by absorbance changes at a wavelength of 340 nm with PK assay kit (Jiancheng Bioengineering Institute, Nanjing, China) and G6PD assay kit (Genmed, Arlington, USA).

### Energy Status

The *ΔΨ* was determined by JC-1 (5, 5′, 6, 6′ - tetrachloro - 1, 1′, 3, 3′ - tetraethylbenzimidazolcarbocyanine iodide) method (Kaiji, Nanjing, China). Cells with a higher *ΔΨ* can absorb more JC-1 into the mitochondria to form polymers, which can be visualized by fluorescence microscopy at 550–620 nm (Nikon, Tokyo, Japan). The content of ATP was measured by the luciferin-luciferase method [14]. Cells were lysed and cleared by centrifugation at 4°C (12,000 g, 5 min), followed by the measurement of the luminescence in a luminometer (Molecular Devices, California, USA).

### Statistic Analysis

Experiments were performed with four replicates, and each experiment was repeated three times. All data were statistically analyzed by ANOVA, and Duncan’s multiple range tests using the SAS software system. *P<*0.05 was considered as a significant difference.

## Results and Discussion

### Effects of Glucose Availability on mRNA Expression of the Key Genes Involved in Milk Fat Synthesis

**Figure 1:**
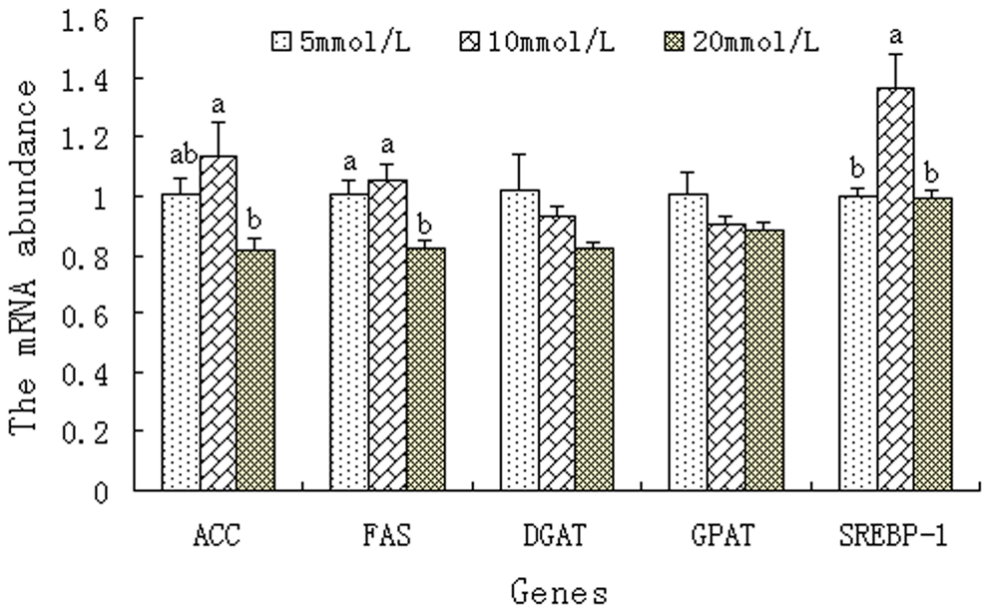
The mRNA expression of key genes involved in milk fat synthesis in BMEC. BMEC were treated for 12 h with various concentration of glucose. Values with different superscripts (a, b) are significantly different (*P*<0.05), and bars indicate the standard error of means (n = 4). BMEC: bovine mammary epithelial cells, ACC: acetyl-CoA carboxylase, FAS: fatty acid synthase; DGAT: diacyl glycerol acyl transferase, GPAT: glycerol-3 phosphate acyl transferase, SREBP-1: sterol regulatory element binding protein-1.

The modification of milk fat production by glucose is a principal aspect of dairy cow nutrition. However, little information is available on how glucose availability affects milk fat synthesis in dairy cows. The synthesis of milk fat may be regulated at multiple levels including transcription, translation, and protein turnover [15], [16]. Acetyl-CoA carboxylase (ACC), fatty acid synthase (FAS), diacyl glycerol acyl transferase (DGAT) and glycerol-3 phosphate acyl transferase (GPAT) are the rate-limiting steps of milk fat synthesis [17], [18]. The genes specifying these enzymes, implicated in the key processes of lipogenesis within the mammary gland, are candidate genes whose regulation has been studied first [16], [19]. In the current study, it was found that compared with 5 mmol/L glucose, higher concentrations of glucose (10–20 mmol/L) did not affect the mRNA abundance of ACC, DGAT and GPAT (p>0.05; Figure 1), while 20 mmlol/L glucose resulted in markedly lower expression of FAS mRNA (*p<*0.05; Figure 1). The sterol regulatory element binding protein-1 (SREBP-1) is the key transcription factor that controls expression of the genes encoding the enzymes for milk fat synthesis [20], [21]. Relative to control (5 mmol/L), SREBP-1 mRNA expression increased at 10 mmol/L and then decreased at 20 mmol/L (*p<*0.05; Figure 1). The increase of SREBP-1 mRNA at 10 mmol/L is thought to be a salvage response, which will increase the expression and activity of proteins required for nutrient acquisition [22], and the specific mechanisms of this response are yet to be studied. The high concentration of glucose decreased relative genes expression involved in milk fat synthesis accompanied with previous findings that duodenal glucose infusions reduced milk fat production due to a decrease of lipoprotein lipase activity and a decrease of intramammary esterification process [5], [23]. Transcription of the genes for lipogenic enzymes is also regulated by glucose in adipose tissue, liver, and pancreatic β-cells [24]. Thus, increasing glucose availability may reduce milk fat synthesis partly by affecting transcription of the lipogenesis genes in the mammary gland.

### Effects of Glucose Availability on mRNA Expression of the Key Genes Involved in Lactose Synthesis and Content of Lactose Synthase

**Figure 2:**
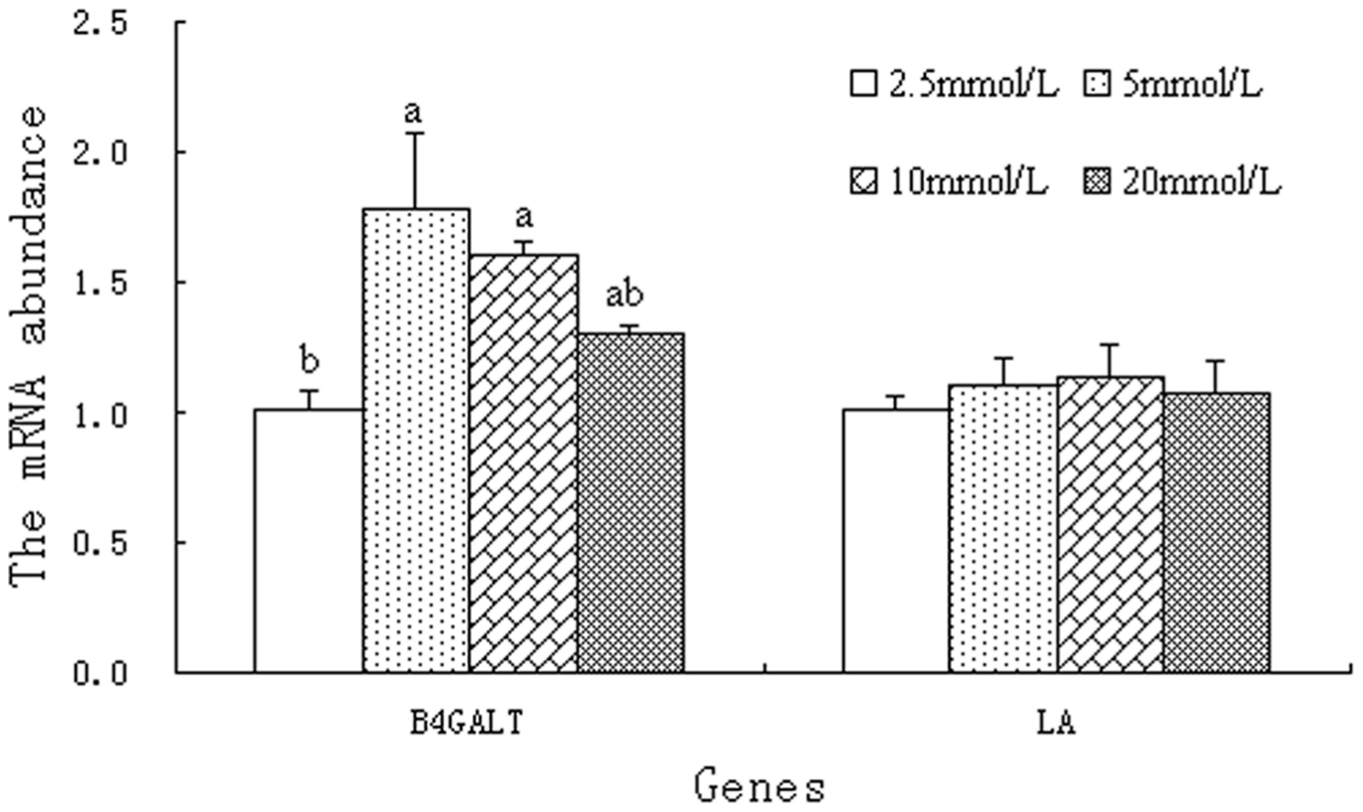
The mRNA expression of key genes involved in lactose synthesis in BMEC. BMEC were treated for 12 h with various concentration of glucose. Values with different superscripts (a, b) are significantly different (*P*<0.05), and bars indicate the standard error of means (n = 4). BMEC: bovine mammary epithelial cells, B4GALT : beta-1, 4-galactosyl transferase, LA: α-lactalbumin.

**Figure 3:**
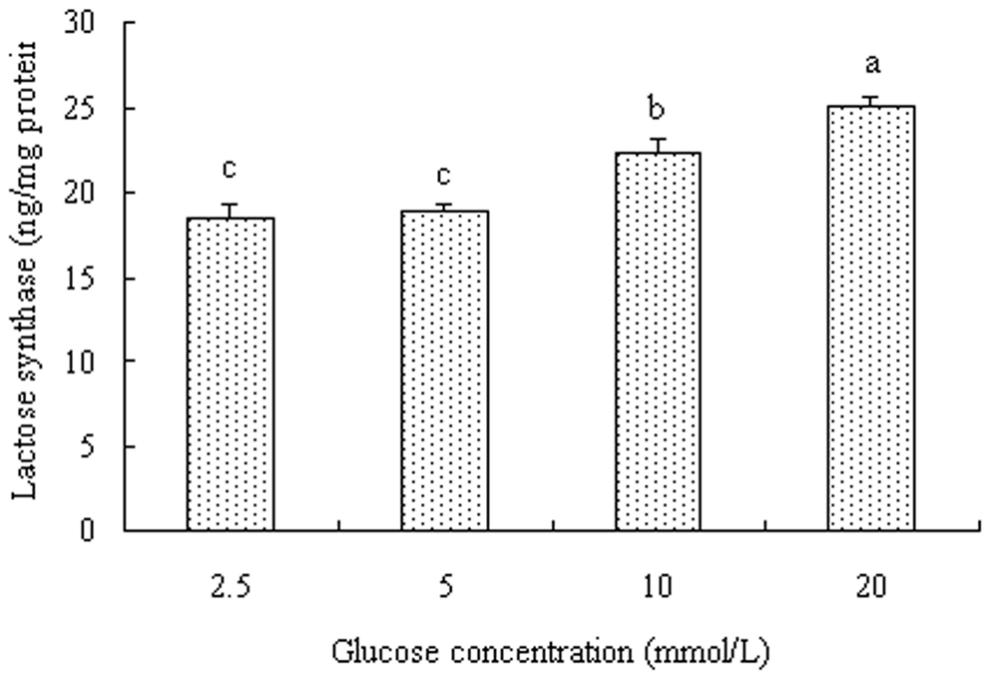
Lactose synthase content in bovine mammary epithelial cells. Bovine mammary epithelial cells were treated for 12 h with various concentration of glucose. Values with different superscripts (a, b) are significantly different (*P*<0.05), and bars indicate the standard error of means (n = 4).

Glucose is the primary precursor of lactose, and its availability is strongly linked to lactose synthesis. However, the mechanism by which increased glucose availability affects lactose synthesis in BMEC is yet unclear. Farrell et al. showed that the membrane-bound enzyme, beta-1, 4-galactosyl transferase (B4GALT) and the milk protein α-lactalbumin (LA) bind to form LS, which synthesizes lactose in the Golgi apparatus of mammary epithelial cells [25]. B4GALT is the only enzyme known to transfer galactose from uridine 5′-diphospho-galactose to terminal N-acetylglucoseamine to form lactose [26]. Compared with the 2.5 mmol/L glucose treatment group, B4GALT mRNA was higher in the 5 and 10 mmol/L glucose treatments but not in the 20 mmol/L treatment (*p<*0.05; Figure 2). In contrast to B4GALT mRNA, no obvious changes of LA mRNA level were found among cells cultured with the various level of glucose (2.5–20 mmol/L) (*p*>0.05; Figure 2). Besides the expression of B4GALT and LA, the content of LS was also evaluated. The LS content was higher in BMEC incubated with 10 and 20 mmol/L glucose than those incubated with 2.5 and 5 mmol/L glucose (*P<*0.05, Figure 3). Increased LS content was most closely correlated with increase of lactose biosynthesis [27], which was thought to be involved in the enhanced milk yield [28]. Based on current literature and our results, increasing glucose availability may stimulate lactose synthesis partly by altering the expression of B4GALT at transcriptional and post-transcriptional levels and then increase milk yield.

### Effects of Glucose Availability on Glucose Metabolism

**Figure 4:**
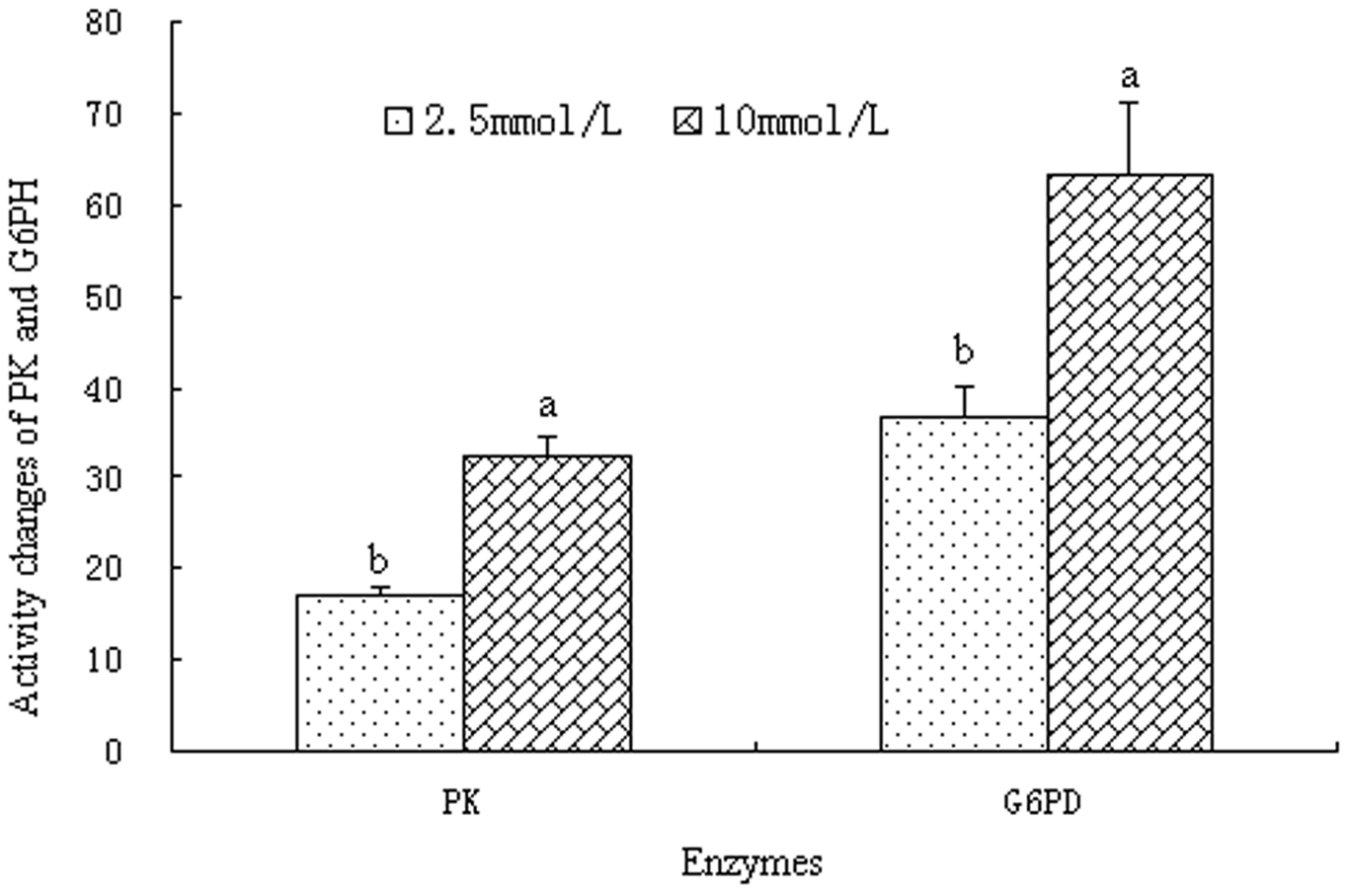
Activity of pyruvate kinase and glucose-6-phosphate dehydrogenase in BMEC. BMEC were treated for 12 h with various concentration of glucose. Values with different superscripts (a, b) are significantly different (*P*<0.05), and bars indicate the standard error of means (n = 4). BMEC: bovine mammary epithelial cells, PK: pyruvate kinase, G6PD: glucose-6-phosphate dehydrogenase.

Glucose in BMEC is mainly used for lactose synthesis [29]. However, metabolism via glycolysis and pentose phosphate pathway also plays an important role in glucose action [30]. Glycolysis is part of a universal metabolic pathway for the catabolic conversion of glucose to energy. Phosphofructokinase, hexokinase, and PK are potential sites of control in the metabolic pathway. PK catalyzes the last step of glycolysis, in which pyruvate and ATP are formed. In the present study, PK activity was increased significantly in the presence of 10 mmol/L glucose compared with 2.5 mmol/L glucose (*P<*0.05, Figure 4). Renner et al. reported that the concentration of glucose influenced glucose metabolic pathways and the relative flux through there pathways in rat hepatoma cells [31]. At low levels (5 to 50 µ*M*), glucose was used by the cells for macromolecular synthesis and oxidative processes. With concentrations higher than 1 mmol/L, glycolysis removed all excess glucose and converted to lactate [31]. The pentose phosphate pathway, which is a process that generates NADPH and pentoses, is an alternative to glycolysis. G6PD is the rate-controlling enzyme of this pathway. In our results, G6PD activity showed a similar trend with PK. It was higher at 10 mmol/L glucose than that at 2.5 mmol/L glucose (*P<*0.05, Figure 4), which was consistent with the findings of Rigout et al. [5]. This result indicates that increases in PK and G6PD activity may increase glucose metabolism by glycolysis and pentose phosphate pathway when BMEC are exposed to elevated glucose concentrations.

**Figure 5:**
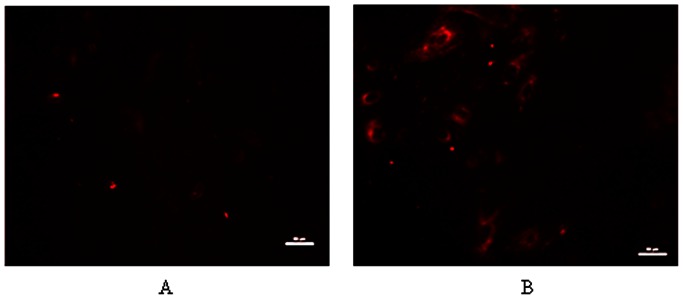
Mitochondrial potential in bovine mammary epithelial cells. Bovine mammary epithelial cells were treated for 12 h with 2.5 mmol/L glucose (A) or 10 mmol/L glucose (B). Bars represent 50 µm.

**Figure 6:**
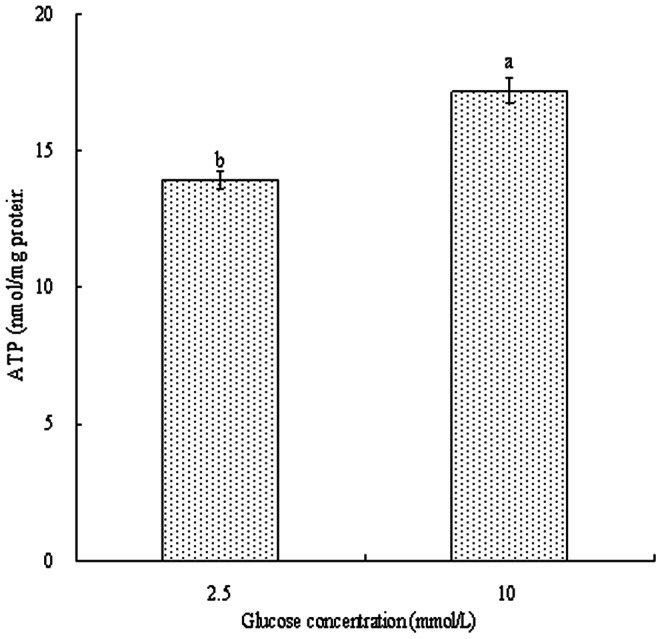
ATP content in bovine mammary epithelial cells. Bovine mammary epithelial cells were treated for 12 h with various concentration of glucose. Values with different superscripts (a, b) are significantly different (*P*<0.05), and bars indicate the standard error of means (n = 6).

The energy status of BMEC was also evaluated. It is well known that milk synthesis is the major part of cellular biosynthesis, which based on the energy supply [32]. Cellular *ΔΨ*, a marker of cell energy status, is a dominant component of respiring mitochondria and directly related to ATP production potential [33]. Greater *ΔΨ* and ATP levels were observed in BMEC incubated with 10 mmol/L glucose than those incubated with 2.5 mmol/L glucose (*P<0.05*, Figures 5 and 6), suggesting that the increased energy supply partly accounted for the enhanced milk synthesis in the cells. It was demonstrated that high glucose levels increased cell *ΔΨ,* and affected energy-dependent cell proliferation of chicken thymocytes and pancreatic cancer cells [34], [35]. Thus, glucose levels regulated the energy status of BMEC, indicating that changes in glucose availability or glucose metabolism may affect energy-dependent processes, such as BMEC proliferation and milk synthesis.

### Conclusions

Compared with physiological concentrations of glucose (2–5 mmol/L) [36], elevated level of glucose may affect the synthesis of milk fat and lactose by regulating the mRNA expression of key genes (ACC, FAS, SREBP-1 and B4GALT), promote glucose metabolism through glycolysis and pentose phosphate pathway and alter the energy status of cells. In conclusion, this study revealed that after increasing glucose availability, the extra glucose absorbed may promote glucose metabolism, and affect the synthesis of milk composition.

## References

[1] Bell AW, Bauman DE (1997) Adaptations of glucose metabolism during pregnancy and lactation. J Mammary Gland Biol Neoplasia 2: 265–278. 10882310 10.1023/a:1026336505343

[2] Annison EF, Linzell JL (1964) The oxidation and utilization of glucose and acetate by the mammary gland of the goat in relation to their over-all metabolism and to milk formation. J Physiol 175: 372–385. 14241838 10.1113/jphysiol.1964.sp007522 PMC1357142

[3] Hurtaud C, Lemosquet S, Rulquin H (2000) Effect of graded duodenal infusions of glucose on yield and composition of milk from dairy cows. 2. Diets based on grass silage. J Dairy Sci 83: 2952–2962. 11132867 10.3168/jds.S0022-0302(00)75195-2

[4] Rulquin H, Rigout S, Lemosquet S, Bach A (2004) Infusion of glucose directs circulating amino acids to the mammary gland in well-fed dairy cows. J Dairy Sci 87: 340–349. 14762077 10.3168/jds.S0022-0302(04)73173-2

[5] Rigout S, Lemosquet S, Van Eys JE, Blum JW, Rulquin H (2002) Duodenal glucose increases glucose fluxes and lactose synthesis in grass silage-fed dairy cows. J Dairy Sci 85: 595–606. 11949864 10.3168/jds.S0022-0302(02)74113-1

[6] Lemosquet S, Rideau N, Rulquin H, Faverdin P, Simon J, et al (1997) Effects of a duodenal glucose infusion on the relationship between plasma concentrations of glucose and insulin in dairy cows. J Dairy Sci 80: 2854–2865. 9406078 10.3168/jds.S0022-0302(97)76250-7

[7] Al-Trad B, Reisberg K, Wittek T, Penner GB, Alkaassem A, et al (2009) Increasing intravenous infusions of glucose improve body condition but not lactation performance in midlactation dairy cows. J Dairy Sci 92: 5645–5658. 19841224 10.3168/jds.2009-2264

[8] Oldick BS, Staples CR, Thatcher WW (1997) Abomasal infusion of glucose and fat-effect on digestion, production, and ovarian and uterine functions of cows. J Dairy Sci 80: 1315–1328. 9241593 10.3168/jds.S0022-0302(97)76060-0

[9] Judson GJ, Leng RA (1973) Studies on the control of gluconeogenesis in sheep: effect of glucose infusion. Brit J Nutr 29: 175–195. 4693554 10.1079/bjn19730092

[10] Xiao CT, Cant JP (2005) Relationship between glucose transport and metabolism in isolated bovine mammary epithelial cells. J Dairy Sci 88: 2794–2805. 16027193 10.3168/jds.S0022-0302(05)72959-3

[11] Zhao K, Liu HY, Wang HF, Zhou MM, Liu JX (2012) Effect of glucose availability on glucose transport in bovine mammary epithelial cells. Animal 6: 488–493. 22436228 10.1017/S1751731111001893

[12] Zhao K, Liu H, Zhou M, Liu J (2010) Establishment and characterization of a lactating bovine mammary epithelial cell model for the study of milk synthesis. Cell Biol Int 34: 717–721. 20214659 10.1042/CBI20100023

[13] Livak KJ, Schmittgen TD (2001) Analysis of relative gene expression data using real-time quantitative PCR and the 2-ΔΔCT method. Methods 25: 402–408. 11846609 10.1006/meth.2001.1262

[14] Kimmich GA, Randles J, Brand JS (1975) Assay of picomole amounts of ATP, ADP, and AMP using the luciferase enzyme system. Anal Biochem 69: 187–206. 2029 10.1016/0003-2697(75)90580-1

[15] Harvatine KJ, Boisclair YR, Bauman DE (2009) Recent advances in the regulation of milk fat synthesis. Animal 3: 40–54. 22444171 10.1017/S1751731108003133

[16] Vaulont S, Vasseur-Cognet M, Kahn A (2000) Glucose regulation of gene transcription. J Biol Chem 275: 31555–315558. 10934218 10.1074/jbc.R000016200

[17] Bionaz M, Loor JJ (2008) Gene networks driving bovine milk fat synthesis during the lactation cycle. BMC Genomics 9: 366–387. 18671863 10.1186/1471-2164-9-366 PMC2547860

[18] Bernard L, Leroux C, Chilliard Y (2008) Expression and nutritional regulation of lipogenic genes in the reminant lactating mammary gland. Adv Exp Med Biol 606: 67–108. 18183925 10.1007/978-0-387-74087-4_2

[19] Towle HC, Kaytor EN, Shih HM (1997) Regulation of the expression of lipogenic enzyme genes by carbohydrate. Annu Rev Nutr 17: 405–433. 9240934 10.1146/annurev.nutr.17.1.405

[20] Edwards PA, Tabor D, Kast HR, Venkateswaran A (2000) Regulation of gene expression by SREBP and SCAP. Bioch et Bioph Acta 1529: 103–113. 10.1016/s1388-1981(00)00140-2 11111080

[21] Bauman DE, Perfield JW, Harvatine KJ, Baumgard LH (2008) Regulation of fat synthesis by conjugated linoleic acid: lactation and the ruminant model. J Nutr 138: 403–409. 18203911 10.1093/jn/138.2.403

[22] Hammerman PS, Fox CCJ, Thompson CB (2004) Beginnings of a signal-transduction pathway for bioenergetic control of cell survival. Trends Biochem Sci 29: 586–592. 15501677 10.1016/j.tibs.2004.09.008

[23] Hurtaud C, Rulquin H, Verite R (1998) Effect of graded duodenal infusions of glucose on yield and composition of milk from dairy cows. 1. Diets based on corn silage. J Dairy Sci 81: 3239–3247. 9891269 10.3168/jds.S0022-0302(98)75888-6

[24] Girard J, Ferré P, Foufelle F (1997) Mechanisms by which carbohydrates regulate expression on genes for glycolytic and lipogenic enzymes. Annu Rev Nutr 17: 325–352. 9240931 10.1146/annurev.nutr.17.1.325

[25] Farrell Jr HM, Jimenez-Flores R, Bleck GT, Brown EM, Butler JE, et al (2004) Nomenclature of the proteins of cows’ milk-sixth revision. J Dairy Sci 87: 1641–1674. 15453478 10.3168/jds.S0022-0302(04)73319-6

[26] Ramakrishnan B, Qasba PK (2001) Crystal structure of lactose synthase reveals a large conformational change in its catalytic component, the β 1, 4-galactosyltransferase-I. J Mol Biol 310: 205–218. 11419947 10.1006/jmbi.2001.4757

[27] Mellenberger RW, Bauman DE (1974) Lactose synthesis in rabbit mammary tissue during pregnancy and lactation. Biochem J 142: 659–665. 4219277 10.1042/bj1420659 PMC1168332

[28] Lemosquet S, Rigout S, Bach A, Rulquin H, Blum JW (2004) Glucose metabolism in lactating cows in response to isoenergetic infusions of propionic acid or duodenal glucose. J Dairy Sci 87: 1767–1777. 15453491 10.3168/jds.S0022-0302(04)73332-9

[29] Kleiber M, Black AL, Brown MA, Baxter CF, Luick JR, et al (1955) Glucose as a precursor of milk constituents in the intact dairy cow. Bioch et Bioph Acta 17: 252–260. 10.1016/0006-3002(55)90357-7 13239666

[30] Abraham S, Hirsch PF, Chaikoff IL (1954) The quantitiative significance of glycolysis and non-glycolysis in glucose utilization by rat mammary gland. J Biol Chem 211: 31–38. 13211638

[31] Renner ED, Plagemann PGW, Bernlohr RW (1972) Permeation of glucose by simple and facilitated diffusion by Novikoff rat hepatoma cells in suspension culture and its relationship to glucose metabolism. J Biol Chem 247: 5765.–5776. 4341490

[32] Möbius K, Arias-Cartin R, Breckau D, Hännig A, Riedmann K, et al (2010) Heme biosynthesis is coupled to electron transport chains for energy generation. Proc Natl Acad Sci USA 107: 10436–10441. 20484676 10.1073/pnas.1000956107 PMC2890856

[33] Rathmell JC, Heiden MGV, Harris MH, Frauwirth KA, Thompson CB (2000) In the absence of extrinsic signals, nutrient utilization by lymphocytes is insufficient to maintain either cell size or viability. Mol Cell 6: 683–692. 11030347 10.1016/s1097-2765(00)00066-6

[34] Humphrey BD, Rudrappa SG (2008) Increased glucose availability activates chicken thymocyte metabolism and survival. J Nutr 138: 1153–1157. 18492849 10.1093/jn/138.6.1153

[35] Han L, Ma Q, Li J, Liu H, Li W, et al (2011) High glucose promotes pancreatic cancer cell proliferation via the induction of EGF expression and transactivation of EGFR. PLoS One 6(11): e27074. 22087246 10.1371/journal.pone.0027074 PMC3210779

[36] Kahn CM (2005) Merck veterinary manual. 9th Ed. Merck & Co., Inc., Whitehouse Station, N.J. 2586 P.

